# Higher-Order Mode Suppression in Antiresonant Nodeless Hollow-Core Fibers

**DOI:** 10.3390/mi10020128

**Published:** 2019-02-15

**Authors:** Aichen Ge, Fanchao Meng, Yanfeng Li, Bowen Liu, Minglie Hu

**Affiliations:** Ultrafast Laser Laboratory, School of Precision Instrument and Optoelectronics Engineering, Key Laboratory of Optoelectronic Information Technology (Ministry of Education), Tianjin University, Tianjin 300072, China; gerchtj@tju.edu.cn (A.G.); mengfc1989@tju.edu.cn (F.M.); huminglie@tju.edu.cn (M.H.)

**Keywords:** hollow-core fiber, antiresonant fiber, negative curvature fiber

## Abstract

Negative curvature hollow-core fibers (NC-HCFs) are useful as gas sensors. We numerically analyze the single-mode performance of NC-HCFs. Both single-ring NC-HCFs and nested antiresonant fibers (NANFs) are investigated. When the size of the cladding tubes is properly designed, higher-order modes (HOMs) in the fiber core can be coupled with the cladding modes effectively and form high-loss supermodes. For the single-ring structure, we propose a novel NC-HCF with hybrid cladding tubes to enable suppression of the first two HOMs in the core simultaneously. For the nested structure, we find that cascaded coupling is necessary to maximize the loss of the HOMs in NANFs, and, as a result, NANFs with five nested tubes have an advantage in single-mode guidance performance. Moreover, a novel NANF with hybrid extended cladding tubes is proposed. In this kind of NANF, higher-order mode extinction ratios (HOMERs) of 10^5^ and even 10^6^ are obtained for the LP_11_ and LP_21_ modes, respectively, and a similar level of 10^5^ for the LP_02_ modes. Good single-mode performance is maintained within a broad wavelength range. In addition, the loss of the LP_01_ modes in this kind of NANF is as low as 3.90 × 10^−4^ dB/m.

## 1. Introduction

Hollow-core (HC) fibers are optical fibers that can confine the main part of the electric field within a hollow core, along which gases or liquids can flow. Their hollow-core microstructure makes these fibers promising tools for optofluidic applications, for example, as gas sensors [1,2]. Meanwhile, the gas-filled HC fiber can be used as a nonlinear medium for supercontinuum [3,4,5] or high-order harmonic generation [6]. The simplest HC fiber is a capillary; however, its high confinement loss is an inherent problem [7,8,9]. Compared with capillaries, the loss of microstructure HC fibers is much lower. Generally, there are two families of microstructure HC fibers: photonic bandgap fibers (HC-PBGFs) and hollow-core antiresonant fibers (HC-ARFs).

An HC-PBGF has an air core in the center with a periodic structure cladding. The light propagating in the core is confined by the photonic bandgap of the periodic cladding structure. As reported in [10], the minimum confinement loss of HC-PBGFs can be as low as 1.2 dB/km. However, the bandwidth of HC-PBGFs is only around 10–30% of the central wavelength [11].

The confinement mechanism of HC-ARFs is different. A series of thin glass walls that function like Fabry–Perot resonators surround the air core in an HC-ARF. This mechanism provides a broad bandwidth for guiding light. In 2017, Wang et al. proposed a clear physical insight into the optical guidance mechanism in HC-ARFs based on a multi-layered model [12]. In the last few years, several types of HC-ARFs have been studied. The first type is Kagome fibers, which consist of multiple layers of thin glass walls and air holes [3]. Kagome fibers have a wide bandwidth that can reach several hundred nanometers and have attracted significant interest. It has been shown that Kagome fibers confine the light mainly by the first glass layer around the air core [13,14], which motivates the following intensive studies on the negative curvature hollow-core fibers (NC-HCFs) [15,16,17,18,19]. The microstructure of NC-HCFs, which typically have one or two thin glass tube claddings, is simpler than that of Kagome fibers. NC-HCFs have been studied widely, and various novel structures have been proposed [11,20,21,22,23,24,25,26,27,28,29]. Because of the unique confinement mechanism of NC-HCFs, they can be designed into a slotted structure [18,28] to allows gases to flow into the hollow-core.

A general problem with NC-HCFs is that most of the time they support not only the fundamental mode but also a family of higher-order modes (HOMs). However, single-mode guidance is required in many applications. For application in gas sensors, HOMs can give rise to modal interference effects that produce complex patterns of oscillations in the transmitted spectrum [1]. Improving the single-mode guidance performance of NC-HCFs is necessary before they can be employed in optofluidic applications. In 2016, Uebel et al. proposed a robust broadband single-mode NC-HCF [15]. The ratio between the diameters of the core and the cladding tubes in their fiber is 0.68. In this way, the LP_11_ modes in the core are coupled with the cladding modes and form high-loss supermodes, which results in high confinement loss of the LP_11_ modes. Broadband phase matching between the LP_11_ and cladding modes provides robust single-mode guidance at all wavelengths within the LP_01_ transmission window. To date, only the phase matching for one single HOM has been investigated, but several HOMs may be stimulated simultaneously in a large-core fiber [30].

Nested antiresonant fibers (NANFs) are NC-HCFs with nested and non-touching antiresonant tube elements arranged around a central core. The confinement loss of the LP_01_ modes can be further reduced owing to the double antiresonant layer. Unfortunately, the losses of HOMs in an NANF will be reduced too. In 2018, L. Provino studied the single-mode guidance performance of NANFs [29]. L. Provino improved the single-mode guidance performance of NANFs by realizing the high-loss supermode of the LP_11_ mode. However, the complex cladding tubes of the NANFs lead to different phase-matching conditions worthy of further investigation.

In this paper, we study the single-mode guidance performance of several types of NC-HCFs based on finite element simulations (COMSOL Multiphysics, COMSOL, Stockholm, Sweden). Not only the LP_11_ modes but also the LP_21_ modes are considered in the suppression of HOMs. To increase the loss of these HOMs, the parameters of the fiber structure are designed to achieve strong coupling between these core modes and cladding modes. The supermodes of the HOMs and cladding modes are high-loss modes; thus, the HOMs are effectively suppressed. The article is structured as follows. Section 2 elucidates the design principles of single tube layer cladding NC-HCFs with high single-mode guidance performance. Then, we propose a novel NC-HCF with hybrid cladding tubes to improve the single-mode guidance performance. In this kind of fiber, high-loss supermodes of both the LP_11_ and the LP_21_ modes are realized simultaneously. Section 3 summarizes the single-mode guidance performance of NANFs. We present the phase-matching condition of NANFs and an LP_11_ mode-suppressed NANF structure. A novel nested antiresonant structure with hybrid extended cladding tubes is described in Section 4. This NANF combines the merits of the NC-HCF with hybrid cladding tubes described in Section 2 with those of the LP_11_ mode-suppressed NANF described in Section 3. It shows excellent single-mode guidance performance. All of the higher-order mode extinction ratios (HOMERs) calculated at 1040 nm are greater than 10^5^. Broadband phase matching for high-loss supermodes is realized. Conclusions are drawn in Section 5.

## 2. Single-Mode Guidance Performance of Single Tube Layer Cladding NC-HCFs

In this paper, the fiber material was assumed to be silica. The cladding tube thickness *t* = 0.248 μm, which is the first antiresonant thickness for a 1040 nm light wave according to the equation [19]:

(1) $$t=\frac{(m-0.5)\lambda }{2\sqrt{n_{\mathrm{silica}}^{2}-n_{\mathrm{air}}^{2}}}$$

where λ is the wavelength and *n*_silica_ and *n*_air_ are the refractive indices of silica and air, respectively. The core diameter *D*_core_ = 20 × 1040 nm. To accurately calculate the confinement loss, a perfectly matched layer was utilized in the outmost boundary of the geometries. A Sellmeier equation was used to describe the dispersion for silica [31]. The material loss was neglected, since the material absorption is quite low in this wavelength range [32]. The refractive index of air was chosen to be 1. The wavelength in this simulation was 1040 nm if not specified otherwise.

**Figure 1:**
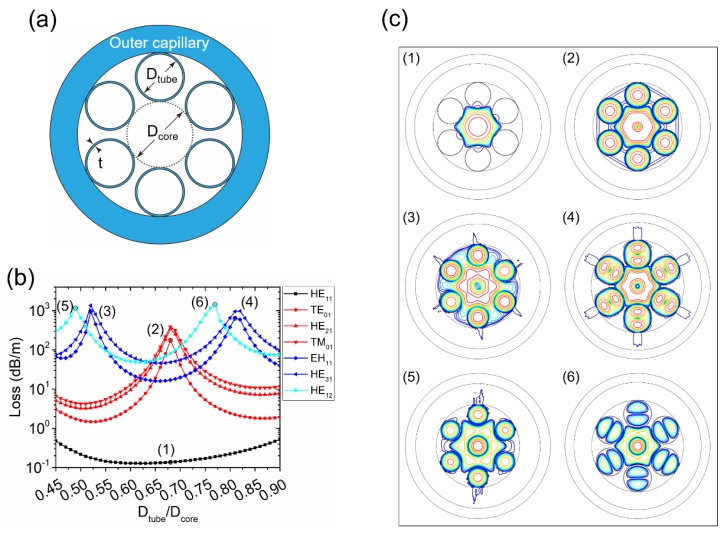
(**a**) Geometry of the six-tube negative curvature hollow-core fiber (NC-HCF) structure. (**b**) Simulated losses of the fundamental HE_11_ mode and six higher-order modes (HOMs) (TE_01_, HE_21_, TM_01_, EH_11_, HE_31_, and HE_12_) as a function of *D*_tube_/*D*_core_. (**c**) Three decibel (3-dB) contour lines of mode patterns: (1) LP_01_ modes at *D*_tube_/*D*_core_ = 0.68, point (1) in (**b**); (2) LP_11_ modes at *D*_tube_/*D*_core_ = 0.68, point (2) in (**b**); (3) LP_21_ modes at *D*_tube_/*D*_core_ = 0.52, point (3) in (**b**); (4) LP_21_ modes at *D*_tube_/*D*_core_ = 0.81, point (4) in (**b**); (5) LP_02_ modes at *D*_tube_/*D*_core_ = 0.49, point (5) in (**b**); and (6) LP_02_ modes at *D*_tube_/*D*_core_ = 0.77, point (6) in (**b**).

Firstly, we study the classical six-tube NC-HCF, whose geometry is shown in Figure 1a [15]. All cladding tubes surrounding the core have an equal diameter *D*_tube_. Figure 1b shows the simulated loss curves of the fundamental LP_01_ (HE_11_) modes and six HOMs (TE_01_, HE_21_, TM_01_, EH_11_, HE_31_, and HE_12_) as a function of *D*_tube_/*D*_core_. The loss of the LP_11_ group of modes (TE_01_, HE_21_, and TM_01_) has a peak at *D*_tube_/*D*_core_ = 0.68, which is the same as in [15]. When *D*_tube_/*D*_core_ = 0.68, the effective refractive index (*n*_eff_) of the LP_11_ modes in the core is equal to that of the fundamental mode in the cladding tube, and, as a result, a high-loss supermode is formed. This could be explained by the equation [15]:

(2) $$\frac{D_{\mathrm{tube}}}{D_{\mathrm{core}}}=\frac{u_{01}}{u_{11}}\frac{f_{\mathrm{core}}}{f_{\mathrm{tube}}}=0.682$$

where the fitting parameters are $f_{\mathrm{core}}=1.077$ for the core and $f_{\mathrm{tube}}=0.991$ for the cladding tube, and *u*_lm_ is the m-th zero of the Bessel function $J_{l}$. The cladding tube diameter has little influence on the loss of the LP_01_ modes because the effective index of the fundamental core mode in the NC-HCF core is larger than that of the fundamental mode in the annular cladding tube when they have the same diameter [19]. Meanwhile, the effective index increases with the diameter of an annular tube but the cladding tubes always have a smaller diameter than the core. As a result, phase matching between the LP_01_ and cladding tube modes occurs only when *D*_tube_ is larger than *D*_core_. However, it cannot happen in an NC-HCF with six cladding tubes. The mode patterns of LP_01_ and LP_11_ modes when *D*_tube_/*D*_core_ = 0.68 are shown in Figure 1c, where a supermode is clearly observed for the LP_11_ modes. LP_21_ (EH_11_ and HE_31_) and LP_02_ (HE_12_) modes both have two high-loss peaks. The first peak, at *D*_tube_/*D*_core_ = 0.52 for the LP_21_ modes and *D*_tube_/*D*_core_ = 0.49 for the LP_02_ modes, is a result of coupling between the respective HOM in the core and the fundamental mode of the cladding tubes. The second peak, which appears at *D*_tube_/*D*_core_ = 0.81 for the LP_21_ modes and *D*_tube_/*D*_core_ = 0.77 for the LP_02_ modes, results from the coupling with the LP_11_ modes of the cladding tubes. The supermodes corresponding to the loss peaks in Figure 1b are also shown in Figure 1c.

**Figure 2:**
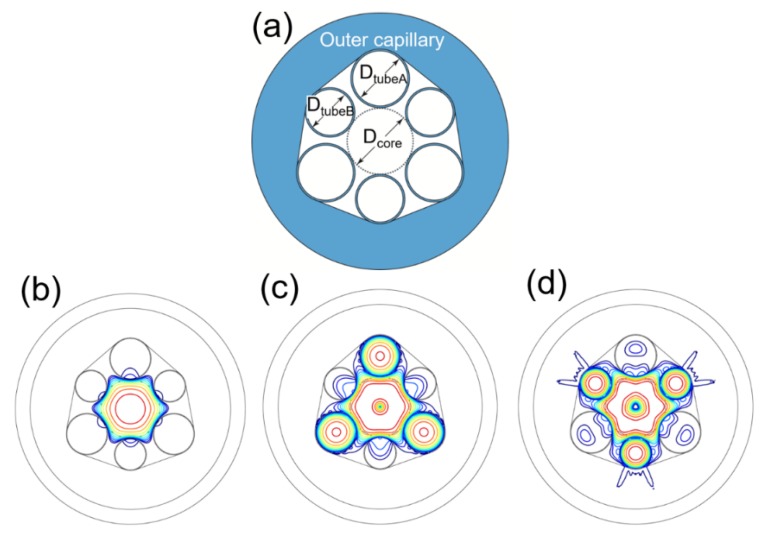
(**a**) Geometry of the hybrid NC-HCF with *D*_tubeA_ = 0.68 *D*_core_ and *D*_tubeB_ = 0.52 *D*_core_. Three decibel (3-dB) contour lines of (**b**) LP_01_ modes, (**c**) LP_11_ modes, and (**d**) LP_21_ modes of the hybrid NC-HCF. Note the similarity with the corresponding mode patters in the top row of Figure 1c.

**Figure 3:**
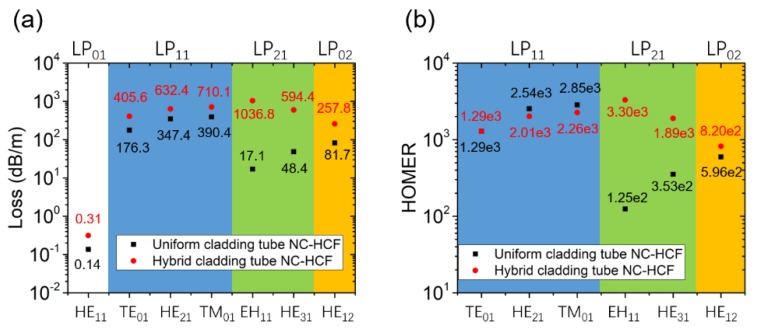
A comparison between the hybrid NC-HCF with *D*_tubeA_ = 0.68 *D*_core_ and *D*_tubeB_ = 0.52 *D*_core_ (red points) and the NC-HCF with uniform cladding tubes with *D*_tube_ = 0.68 *D*_core_ (black squares). (**a**) The confinement loss in units of dB/m and (**b**) the higher-order mode extinction ratio (HOMER).

The first HOM that could be excited in an NC-HCF is the LP_11_ modes, which means that applying cladding tubes with a diameter *D*_tube_ = 0.68 *D*_core_ in an NC-HCF could improve the single-mode guidance performance of the fiber. Although an NC-HCF with *D*_tube_/*D*_core_ = 0.68 can suppress the LP_11_ modes of the fiber, the LP_21_ modes are not strongly suppressed. The loss of the EH_11_ mode is only 17.1 dB/m when *D*_tube_ = 0.68 *D*_core_. To effectively increase the loss of the LP_21_ modes, we propose a novel NC-HCF with hybrid cladding tubes, as shown in Figure 2a. Three of the cladding tubes have diameter *D*_tubeA_ = 0.68 *D*_core_, and the others have diameter *D*_tubeB_ = 0.52 *D*_core_. Different cladding tubes are placed alternately around the fiber core. The inner boundary of the outer capillary is a polygon with rounded corners that closely fits the adjacent cladding tubes. From the analysis above, it is not surprising to see that both the LP_11_ and LP_21_ modes form high-loss supermodes, as verified in Figure 2b–d. The higher-order mode extinction ratio (HOMER) is commonly used to quantitatively describe the single-mode performance [23]. The HOMER is defined as the ratio of the loss of the higher-order core modes to that of the fundamental mode. For comparison, the loss and HOMER of the hybrid NC-HCF and those of the corresponding NC-HCF with uniform cladding tubes (*D*_tube_ = 0.68 *D*_core_) are shown in Figure 3.

The losses and effective refractive indexes of the fiber for the two polarizations were calculated. There is no obvious difference in the fiber mode indices in the two polarizations. In both the vertical and horizontal polarizations in Figure 2a, the loss of the LP_01_ mode is 0.31 dB/m and the effective refractive index is 0.999374, which means that there is no birefringence in the hybrid NC-HCF. The outer capillary of the hybrid cladding tube NC-HCF leads to a higher loss for all the modes. Nevertheless, both the LP_11_ and LP_21_ modes can be coupled to the cladding modes effectively and form high-loss supermodes, which allow their HOMERs to reach a level of 1000. The HOMERs of the LP_11_ modes in the hybrid NC-HCF are close to those of the uniform structure. However, the HOMERs of the LP_21_ modes in the hybrid NC-HCF are notably larger than those of the uniform structure because phase matching for the LP_21_ modes is also achieved in the hybrid NC-HCF. Neither of the two fibers can achieve efficient phase matching for the LP_02_ mode, so they have close HOMER values. As a result of applying the hybrid cladding tubes, the fiber can suppress the LP_11_ and LP_21_ modes simultaneously.

## 3. Single-Mode Guidance Performance of NANFs

**Figure 4:**
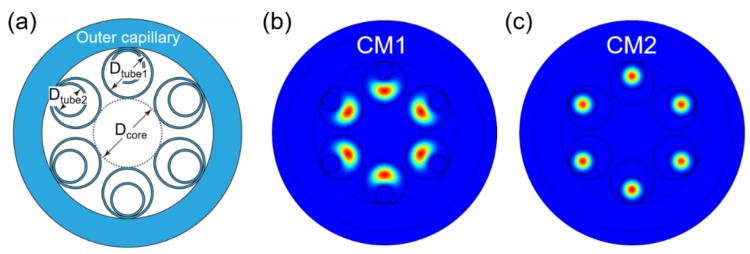
(**a**) Geometry of a nested antiresonant fiber (NANF) with six nested tubes, (**b**) the mode pattern of cladding mode CM1, and (**c**) the mode pattern of cladding mode CM2.

Compared with NC-HCFs, which have only one antiresonant layer, NANFs could reduce not only the confinement loss of the LP_01_ fundamental modes but also that of the HOMs. The geometry of a typical NANF structure, consisting of six nested tubes, is shown in Figure 4a. Compared with a single antiresonant layer NC-HCF, the phase-matching scenario for high-loss supermodes in an NANF is more complicated. Two cladding modes (CM1 and CM2) are typically involved in the phase matching. CM1 is located in the middle of the first and second cladding tubes, as shown in Figure 4b. CM2 is located inside the second cladding tube, as shown in Figure 4c.

**Figure 5:**
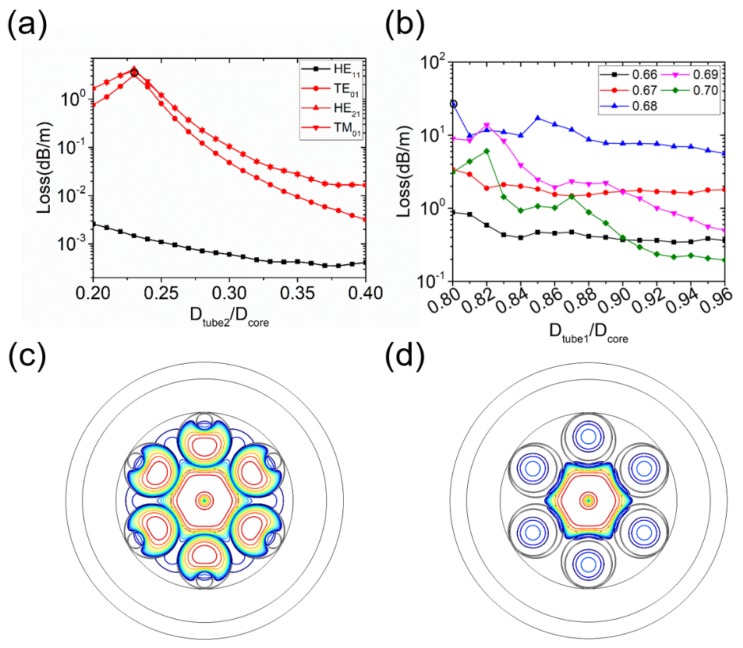
(**a**) The loss of LP_11_ modes as a function of *D*_tube2_/*D*_core_ in an NANF with six nested tubes when the first cladding tube diameter is fixed at *D*_tube1_ = 0.80 *D*_core_; (**b**) the loss of the LP_11_ (TE_01_) mode as a function of *D*_tube1_/*D*_core_ in an NANF with six nested tubes when the first cladding tube diameter is fixed at *D*_tube2_/*D*_core_ = 0.66, 0.67, 0.68, 0.69, and 0.70; (**c**) 3-dB contour lines of the high-loss LP_11_ supermode when *D*_tube1_/*D*_core_ = 0.80 and *D*_tube2_/*D*_core_ = 0.23; and (**d**) 3-dB contour lines of the high-loss LP_11_ supermode when *D*_tube1_/*D*_core_ = 0.80 and *D*_tube2_/*D*_core_ = 0.68.

To explore the phase matching between the LP_11_ and CM1 modes, we keep the first cladding tube diameter at *D*_tube1_ = 0.80 *D*_core_ and change the second cladding tube diameter [29]. The loss of the LP_11_ modes as a function of *D*_tube2_/*D*_core_ is shown in Figure 5a and reaches a maximum at *D*_tube2_/*D*_core_ = 0.23. However, the loss of the LP_01_ modes increases as the value of *D*_tube2_/*D*_core_ is reduced and the second cladding tube prevents the LP_11_ supermode from reaching a high loss, as can be seen from the mode pattern in Figure 5c. When *D*_tube1_ = 0.80 *D*_core_ and *D*_tube2_ = 0.23 *D*_core_, the loss of the LP_01_ modes is 1.47 × 10^−3^ dB/m, which is quite high for NANFs, while the loss of the LP_11_ modes is no more than 5 dB/m.

It is more difficult to realize phase matching between the LP_11_ and CM2 modes. Because CM2 is a circular tube mode, the phase-matching condition for a high-loss supermode should be close to *D*_tube2_ = 0.68 *D*_core_. We therefore sweep *D*_tube1_ for *D*_tube2_/*D*_core_ = 0.66, 0.67, 0.68, 0.69, and 0.70. The results are shown in Figure 5b. The maximum loss of the LP_11_ mode is 26.9 dB/m and appears at *D*_tube1_ = 0.80 *D*_core_ and *D*_tube2_ = 0.68 *D*_core_, simply the smallest *D*_tube1_ with *D*_tube2_ = 0.68 *D*_core_ in our sweep range. The mode pattern of this supermode is displayed in Figure 5d. The LP_01_ mode loss of this structure is as high as 5.85 × 10^−3^ dB/m. Whether the LP_11_ modes are coupled with CM1 or CM2, their loss cannot be raised to more than 100 dB/m, which can be realized in NC-HCFs with one antiresonant layer. At the same time, the loss of the LP_01_ mode is too high. It is not effective to improve the single-mode guidance performance by coupling the HOM only with one single cladding mode in an NANF if a low loss of the LP_01_ mode is desired.

**Figure 6:**
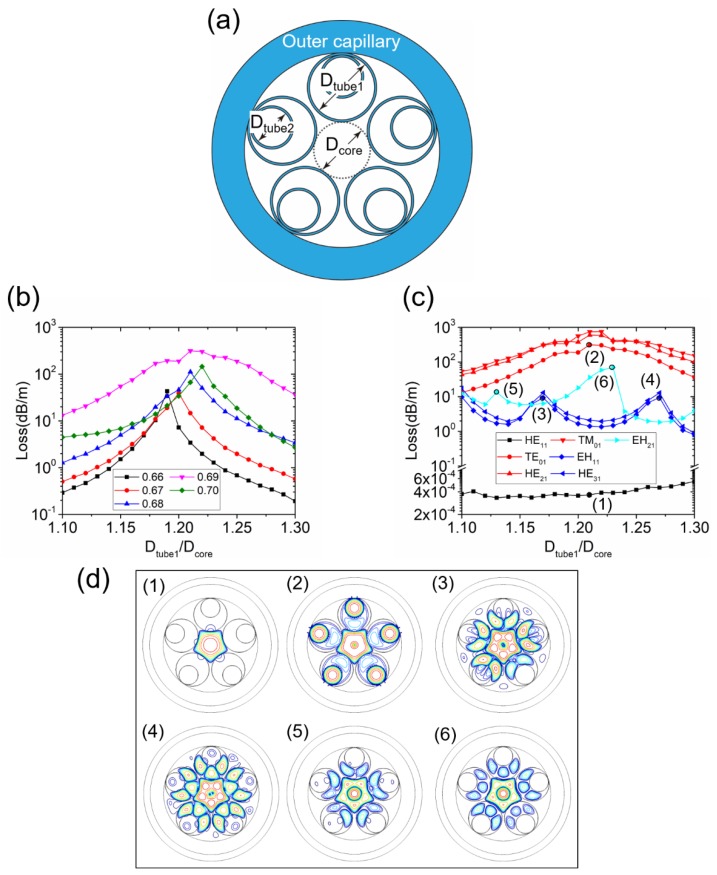
(**a**) Geometry of the NANF with five nested tubes. (**b**) The LP_11_ (TE_01_) mode loss as a function of *D*_tube1_/*D*_core_ for *D*_tube2_/*D*_core_ = 0.66, 0.67, 0.68, 0.69, and 0.70. (**c**) Simulated losses of the fundamental HE_11_ mode and six HOMs (TE_01_, HE_21_, TM_01_, EH_11_, HE_31_, and HE_12_) as a function of *D*_tube1_/*D*_core_ in the NANF with five nested tubes when *D*_tube2_/*D*_core_ = 0.69. (**d**) Three decibel (3-dB) contour lines of the mode patterns when *D*_tube2_/*D*_core_ = 0.69: (1) LP_01_ modes when *D*_tube1_/*D*_core_ = 1.21, point (1) in (**c**); (2) LP_11_ modes when *D*_tube1_/*D*_core_ = 1.21, point (2) in (**c**); (3) LP_21_ modes when *D*_tube1_/*D*_core_ = 1.17, point (3) in (**c**); (4) LP_21_ modes when *D*_tube1_/*D*_core_ = 1.27, point (4) in (**c**); (5) LP_02_ modes when *D*_tube1_/*D*_core_ = 1.13, point (5) in (**c**); and (6) LP_02_ modes when *D*_tube1_/*D*_core_ = 1.23, point (6) in (**c**).

To further increase the loss of the LP_11_ modes in the NANFs and meanwhile maintain a low loss of the LP_01_ modes, we turn to a structure with five nested tubes, as illustrated in Figure 6a. In this way, the area of the cladding modes becomes larger, and, as a result, the HOMs can be coupled with the cladding modes more strongly. A structure with fewer nested tubes could have a larger *D*_tube1_, which makes it possible to realize phase matching between the LP_11_, CM1, and CM2 modes. The losses of the LP_11_ modes as a function of *D*_tube1_/*D*_core_ for *D*_tube2_/*D*_core_ = 0.66, 0.67, 0.68, 0.69, and 0.70 are shown in Figure 6b. An interesting result is that for each *D*_tube2_, the maximum loss value appears when *D*_tube1_/*D*_tube2_ is close to 1.75. This result indicates that *D*_tube1_/*D*_tube2_ = 1.75 fulfils the phase-matching condition between CM1 and CM2. The mode loss is higher when *D*_tube2_ = 0.69 *D*_core_. The maximum loss of the LP_11_ mode is 313.96 dB/m when *D*_tube1_ = 1.21 *D*_core_ and *D*_tube2_ = 0.69 *D*_core_, and the mode pattern is shown in Figure 6d. The loss of the LP_01_ modes is only 3.64 × 10^−4^ dB/m. Thus, the cascaded coupling between the LP_11_, CM1, and CM2 modes increases the LP_11_ mode loss significantly. However, the LP_21_ and LP_02_ modes have different phase-matching conditions. We then keep *D*_tube2_ = 0.69 *D*_core_ and sweep *D*_tube1_ to study the loss of the other modes. As displayed in Figure 6c, similar to the case in a single tube layer cladding NC-HCF, both LP_21_ and LP_02_ modes have two high-loss peaks that correspond to different HOMs of CM1. The high-loss peaks of the LP_21_ modes appear at *D*_tube1_ = 1.17 *D*_core_ and *D*_tube1_ = 1.27 *D*_core_, and the high-loss peaks of the LP_02_ modes appear at *D*_tube1_ = 1.13 *D*_core_ and *D*_tube1_ = 1.23 *D*_core_.

## 4. NANFs with Extended Cladding Tubes

**Figure 7:**
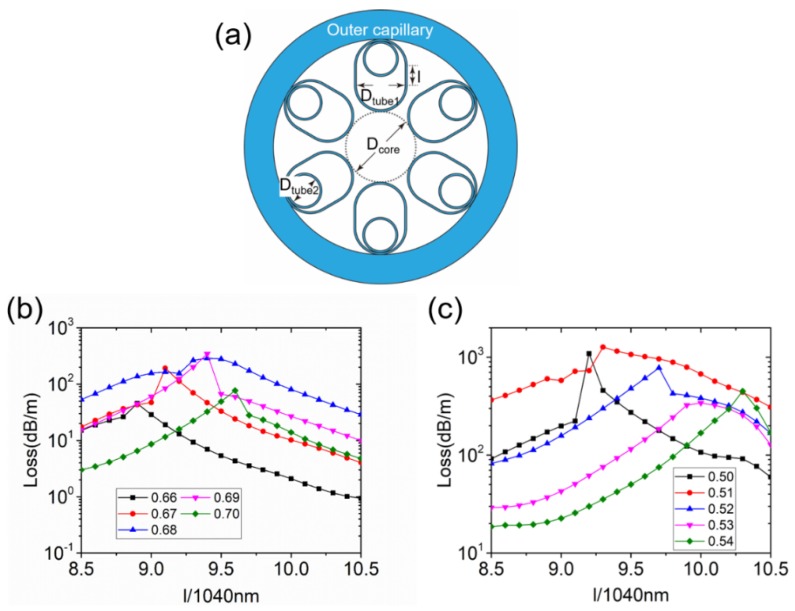
(**a**) Geometry of the NANF with extended cladding tubes. (**b**) The LP_11_ mode loss in the NANF with extended cladding tubes as a function of *l* when *D*_tube2_/*D*_core_ = 0.66, 0.67, 0.68, 0.69, and 0.70. (**c**) The LP_21_ mode loss in the NANF with extended cladding tubes as a function of *l* when *D*_tube2_/*D*_core_ = 0.50, 0.51, 0.52, 0.53, and 0.54.

As illuminated in Section 3, the cascaded coupling between the HOMs in the fiber core and the cladding modes can raise the loss of the HOMs by increasing the area of the cladding modes. It is impossible to realize the cascaded coupling in NANFs with six nested circular cladding tubes. It is also impossible to alternately place the hybrid claddings tubes in an NANF with five nested tubes. To increase the area of CM1 in an NANF with six nested tubes, we propose a novel NANF with extended cladding tubes with the geometry shown in Figure 7a. The first cladding tube is extended with a length of *l*. To simplify the design process, we keep *D*_tube1_ = 0.80 *D*_core_.

**Figure 8:**
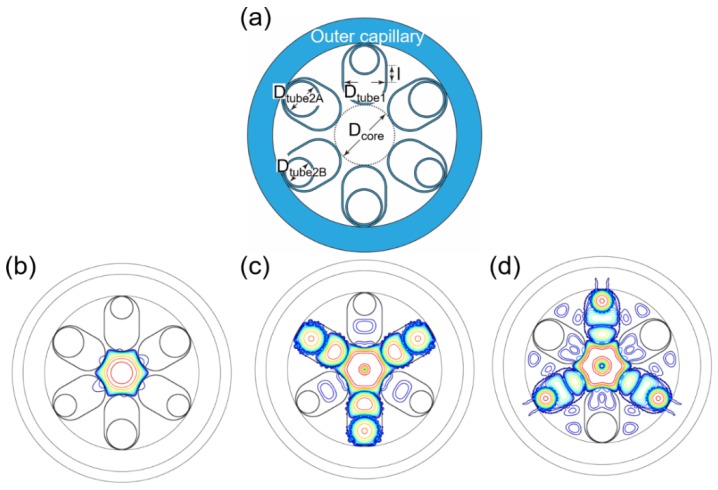
(**a**) Geometry of the NANF with hybrid extended cladding tubes. Three decibel (3-dB) contour plots of (**b**) LP_01_ modes, (**b**) LP_11_ modes, and (**c**) LP_21_ modes of the NANF with hybrid extended cladding tubes.

The loss of the LP_11_ modes in the NANF with extended cladding tubes is investigated as a function of *l* when *D*_tube2_/*D*_core_ = 0.66, 0.67, 0.68, 0.69, and 0.70, as shown in Figure 7b. The maximum loss value is 343.7 dB/m when *l* = 9.4 × 1040 nm and *D*_tube2_/*D*_core_ = 0.69. On the other hand, the LP_21_ mode loss as a function of *l* when *D*_tube2_/*D*_core_ = 0.50, 0.51, 0.52, 0.53, and 0.54 is shown in Figure 7c. The maximum loss value is 1270.3 dB/m when *l* = 9.3 × 1040 nm and *D*_tube2_/*D*_core_ = 0.51. Interestingly, the loss peaks of these two HOMs are close to each other. The second-largest value of the LP_21_ mode loss is 1153.3 dB/m when *l* = 9.4 × 1040 nm and *D*_tube2_/*D*_core_ = 0.51. As a result, it is very easy to apply a hybrid cladding tube structure in NANFs with extended cladding tubes. The geometry of the NANF with hybrid extended cladding tubes is shown in Figure 8a. All of the first cladding tubes have an extended length *l* = 9.4 × 1040 nm. Three of the second cladding tubes have an inner diameter of *D*_tube2A_ = 0.69 *D*_core_ and the others an inner diameter of *D*_tube2B_ = 0.51 *D*_core_. The nested cladding tubes are alternately arranged. The mode patterns of the LP_01_, LP_11_, and LP_21_ modes are shown in Figure 8b–d, respectively. As can be clearly observed, high-loss supermodes are realized for both the LP_11_ and LP_21_ modes.

**Figure 9:**
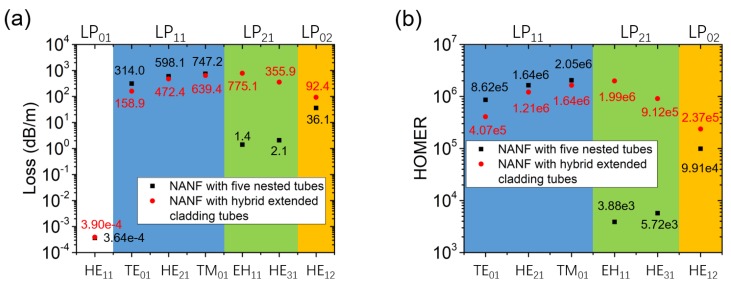
A comparison of (**a**) the loss and (**b**) the HOMER between an NANF with hybrid extended cladding tubes of parameters *D*_tube1_ = 0.80 *D*_core_, *D*_tube2A_ = 0.69 *D*_core_, *D*_tube2B_ = 0.51 *D*_core_ and *l* = 9.4 × 1040 nm (red points) and an NANF with five nested tubes of parameters *D*_tube1_ = 1.21 *D*_core_ and *D*_tube2_ = 0.69 *D*_core_ (black squares).

As for the hybrid NC-HCF, the losses and effective refractive indexes of the fiber in the two polarizations were calculated. In the vertical polarization in Figure 8a, the LP_01_ mode loss is 3.90 × 10^−4^ dB/m and is 4.05 × 10^−4^ dB/m in the horizontal polarization. In both polarizations, the effective refractive index is 0.999363, which means that there is no birefringence in the NANF with hybrid extended cladding tubes. Then, we made a comparison between the two HOM-suppressed NANFs in Figure 9. One structure considered is an NANF with five nested tubes of parameters *D*_tube1_ = 1.21 *D*_core_ and *D*_tube2_ = 0.69 *D*_core_, the circular tube NANF with the best single-mode guidance performance in Section 3. The other structure is an NANF with hybrid extended cladding tubes proposed in this Section. The LP_01_ mode loss of the NANF with five nested tubes is 3.64 × 10^−4^ dB/m. The NANF with hybrid extended cladding tubes has a slightly higher LP_01_ mode loss of 3.90 × 10^−4^ dB/m. The NANF with five nested tubes has a higher LP_11_ mode loss. Although the NANF with five nested tubes has a HOMER of the level of 10^3^ for the LP_21_ modes, the NANF with hybrid extended cladding tubes can achieve a level of 10^5^ or even 10^6^ for the LP_21_ modes because of its extremely high LP_21_ mode loss. A HOMER level of 10^5^ for the LP_20_ modes is also obtained for the NANF with hybrid extended cladding tubes. Thus, the NANF with hybrid extended cladding tubes overall permits a more effective single-mode guidance performance.

**Figure 10:**
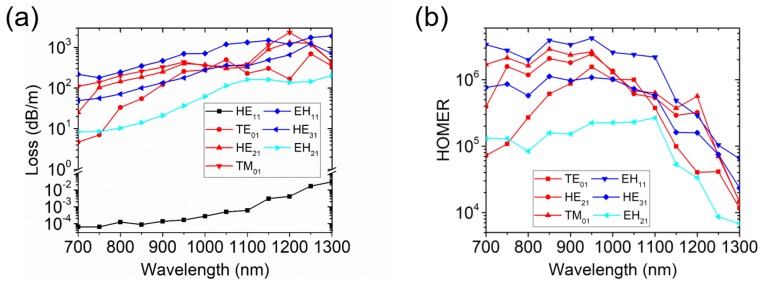
(**a**) The losses of the fundamental mode and HOMs as a function of wavelength in an NANF with hybrid extended cladding tubes; (**b**) The HOMERs of the fundamental mode and HOMs as a function of wavelength in an NANF with hybrid extended cladding tubes.

In the end, we verified the broadband guidance of the NANF with hybrid extended cladding tubes. The loss and the HOMER as a function of wavelength in the range 700–1300 nm are displayed in Figure 10. The losses increase at long wavelengths because the ratio between the core diameter and wavelength becomes lower. A relatively large core could reduce the loss. Similar to the nested elliptical cladding tube NANF in [23], the loss of the fundamental mode of the NANF with hybrid extended cladding tubes rises quickly at long wavelengths. As a result, the HOMERs are reduced at long wavelengths. Phase matching for the LP_11_ and LP_21_ modes are achieved in the wavelength range, so the HOMERs are higher than 10^4^ at most wavelengths. At shorter wavelengths, the HOMERs are typically above 10^5^.

## 5. Conclusions

In conclusion, we have numerically analyzed the single-mode guidance performance of NC-HCFs. The loss of HOMs can be increased by enabling phase matching with cladding modes. To improve the LP_21_ mode loss of single tube layer NC-HCFs, we propose a novel NC-HCF with hybrid cladding tubes. Both the LP_11_ and LP_21_ modes have an HOMER level of 1000 in the hybrid NC-HCF. Phase matching between the LP_11_ and cladding modes in NANFs is also explored. The cascaded coupling between the LP_11_, CM1, and CM2 modes improves the LP_11_ mode loss. High-loss supermodes could be realized in an NANF with five instead of six nested tubes. A novel NANF with hybrid extended cladding tubes is proposed, where not only the LP_11_ but also the LP_21_ modes are suppressed while the loss of the LP_01_ modes is only 3.90 × 10^−4^ dB/m. All of the HOMERs calculated at 1040 nm are larger than 10^5^. Phase matching for high-loss supermodes is realized within a broad band. We believe that the results presented here are of value for further NC-HCF designs and the proposed structures will enable novel applications.

## References

[1] Petrovich M.N., Poletti F., Richardson D.J. Analysis of modal interference in photonic bandgap fibres. Proceedings of the 12th International Conference on Transparent Optical Networks.

[2] Yang F., Jin W., Lin Y., Wang C., Ho H.L., Tan Y. (2017). Hollow-core microstructured optical fiber gas sensors. J. Lightwave Technol..

[3] Travers J.C., Chang W., Nold J., Joly N.Y., Russell P.S.J. (2011). Ultrafast nonlinear optics in gas-filled hollow-core photonic crystal fibers [Invited]. J. Opt. Soc. Am. B.

[4] Hasan M.I., Akhmediev N., Chang W. (2016). Mid-infrared supercontinuum generation in supercritical xenon-filled hollow-core negative curvature fibers. Opt. Lett..

[5] Meng F., Liu B., Wang S., Liu J., Li Y., Wang C., Zheltikov A.M., Hu M. (2017). Controllable two-color dispersive wave generation in argon-filled hypocycloid-core kagome fiber. Opt. Express.

[6] Heckl O.H., Baer C.R.E., Krankel C., Marchese S.V., Schapper F., Holler M., Sudmeyer T., Robinson J.S., Tisch J.W.G., Couny F. (2009). High harmonic generation in a gas-filled hollow-core photonic crystal fiber. Appl. Phys. B.

[7] Nisoli M., De Silvestri S., Svelto O. (1996). Generation of high energy 10 fs pulses by a new pulse compression technique. Appl. Phys. Lett..

[8] Durfee C.G., Backus S., Kapteyn H.C., Murnane M.M. (1999). Intense 8-fs pulse generation in the deep ultraviolet. Opt. Lett..

[9] Popmintchev T., Chen M.-C., Arpin P., Murnane M.M., Kapteyn H.C. (2010). The attosecond nonlinear optics of bright coherent x-ray generation. Nat. Photonics.

[10] Roberts P.J., Couny F., Sabert H., Mangan B.J., Williams D.P., Farr L., Mason M.W., Tomlinson A., Birks T.A., Knight J.C. (2005). Ultimate low loss of hollow-core photonic crystal fibres. Opt. Express.

[11] Poletti F. (2014). Nested antiresonant nodeless hollow core fiber. Opt. Express.

[12] Wang Y., Ding W. (2017). Confinement loss in hollow-core negative curvature fiber: A multi-layered model. Opt. Express.

[13] Pearce G.J., Wiederhecker G.S., Poulton C.G., Burger S., Russell P.S.J. (2007). Models for guidance in kagome-structured hollow-core photonic crystal fibres. Opt. Express.

[14] Février S., Beaudou B., Viale P. (2010). Understanding origin of loss in large pitch hollow-core photonic crystal fibers and their design simplification. Opt. Express.

[15] Uebel P., Günendi M.C., Frosz M.H., Ahmed G., Edavalath N.N., Ménard J.-M., Russell P.S.J. (2016). Broadband robustly single-mode hollow-core PCF by resonant filtering of higher-order modes. Opt. Lett..

[16] Liu X., Ding W., Wang Y.Y., Gao S., Cao L., Feng X., Wang P. (2017). Characterization of a liquid-filled nodeless anti-resonant fiber for biochemical sensing. Opt. Lett..

[17] Kosolapov A.F., Alagashev G.K., Kolyadin A.N., Pryamikov A.D., Biryukov A.S., Bufetov I.A., Dianov E.M. (2016). Hollow-core revolver fibre with a double-capillary reflective cladding. Quantum Electron..

[18] Belardi W. (2015). Design and properties of hollow antiresonant fibers for the visible and near infrared spectral range. J. Lightwave Technol..

[19] Wei C., Weiblen R.J., Menyuk C.R., Hu J. (2017). Negative curvature fibers. Adv. Opt. Photonics.

[20] Wei C., Menyuk C.R., Hu J. (2016). Impact of cladding tubes in chalcogenide negative curvature fibers. IEEE Photonics J..

[21] Weiblen R.J., Menyuk C.R., Gattass R.R., Shaw L.B., Sanghera J.S. (2016). Fabrication tolerances in As_2_S_3_ negative-curvature antiresonant fibers. Opt. Lett..

[22] Chaudhuri S., Putten L.V., Poletti F., Sazio P.J.A. (2016). Low loss transmission in negative curvature optical fibers with elliptical capillary tubes. J. Lightwave Technol..

[23] Meng F., Liu B., Li Y., Wang C., Hu M. (2017). Low loss hollow-core antiresonant fiber with nested elliptical cladding elements. IEEE Photonics J..

[24] Yu T., Liu X., Fan Z.W. (2017). Hollow core antiresonant fiber with radially asymmetric nodeless claddings. IEEE Photonics J..

[25] Hasan M.I., Akhmediev N., Chang W. (2017). Positive and negative curvatures nested in an antiresonant hollow-core fiber. Opt. Lett..

[26] Chen Y., Saleh M.F., Joly N.Y., Biancalana F. (2017). Low-loss single-mode negatively curved square-core hollow fibers. Opt. Lett..

[27] Gao S., Wang Y., Ding W., Jiang D., Gu S., Zhang X., Wang P. (2018). Hollow-core conjoined-tube negative-curvature fibre with ultralow loss. Nat. Commun..

[28] Hao Y., Xiao L., Benabid F. (2018). Optimized design of unsymmetrical gap nodeless hollow core fibers for optofluidic applications. J. Lightwave Technol..

[29] Provino L. (2018). Effect of nested elements on avoided crossing between the higher-order core modes and the air-capillary modes in hollow-core antiresonant optical fibers. Fibers.

[30] Gong M., Liao S., Yuan Y., Zhang H. (2009). High-order modes suppression in large-mode-area fiber amplifiers and lasers by controlling the mode power allocations. J. Opt. A Pure Appl. Opt..

[31] Fleming J.W. (1984). Dispersion in GeO_2_–SiO_2_ glasses. Appl. Opt..

[32] Kitamura R., Pilon L., Jonasz M. (2007). Optical constants of silica glass from extreme ultraviolet to far infrared at near room temperature. Appl. Opt..

